# Supplementary material for: A Likelihood Approach for Real-Time Calibration of Stochastic Compartmental Epidemic Models
Source: PLoS Comput Biol. 2017 Jan 17;13(1):e1005257. doi: 10.1371/journal.pcbi.1005257 (PMC5240920; doi:10.1371/journal.pcbi.1005257)
Supplement: S1 File — (TAR.GZ) [file pcbi.1005257.s014.tar.gz › HSPH_Online-SI-Revision/MSS10/n1-severe/n1-severe_NEW2_table-8.pdf]

1	newly 3 weeks spec	19.	29.43	{0., 72.}	122.158	0.802064	newly 3 weeks cumul	34.	91.66	{6., 196.}	200.118	0.499755	newly 5 weeks spec	25.	20.63	{0., 54.}	79.32	1.02674	newly 5 weeks cumul	86.	135.72	{6., 270.}	106.907	0.459315	newly 62 weeks cumul	592.	215.46	{6., 489.}	66.1655	0.770281
2	newly 3 weeks spec	7.	43.05	{6., 93.}	529.286	0.752077	newly 3 weeks cumul	19.	98.74	{24., 201.}	425.684	0.630319	newly 5 weeks spec	33.	53.54	{2., 99.}	97.6364	0.513888	newly 5 weeks cumul	67.	203.95	{38., 389.}	220.343	0.483811	newly 64 weeks cumul	626.	604.14	{65., 865.}	35.7188	0.285954
3	newly 3 weeks spec	17.	22.78	{0., 56.}	122.941	0.96532	newly 3 weeks cumul	50.	61.39	{3., 127.}	88.5	0.48991	newly 5 weeks spec	23.	20.44	{0., 68.}	103.217	1.27644	newly 5 weeks cumul	88.	106.06	{3., 255.}	96.3409	0.594437	newly 63 weeks cumul	618.	163.46	{3., 388.}	73.6084	1.04422
4	newly 3 weeks spec	5.	27.75	{2., 60.}	475.4	0.685542	newly 3 weeks cumul	13.	50.46	{7., 100.}	306.615	0.511705	newly 5 weeks spec	23.	53.72	{3., 111.}	169.652	0.529379	newly 5 weeks cumul	59.	146.26	{14., 286.}	179.763	0.452703	newly 66 weeks cumul	656.	774.57	{552., 922.}	31.9832	0.21272
5	newly 3 weeks spec	21.	14.3	{1., 31.}	56.	0.502569	newly 3 weeks cumul	47.	28.76	{6., 61.}	51.3617	0.39988	newly 5 weeks spec	31.	30.19	{2., 78.}	74.7419	0.545493	newly 5 weeks cumul	110.	79.78	{8., 182.}	56.6	0.426478	newly 65 weeks cumul	613.	683.15	{42., 893.}	35.3915	0.290352
6	newly 3 weeks spec	13.	8.31	{1., 19.}	58.6923	0.49806	newly 3 weeks cumul	22.	19.64	{4., 39.}	56.4545	0.326225	newly 5 weeks spec	15.	13.65	{1., 30.}	70.2	0.531073	newly 5 weeks cumul	45.	44.21	{8., 92.}	62.7333	0.351861	newly 62 weeks cumul	650.	530.42	{195., 760.}	27.3446	0.264842
7	newly 3 weeks spec	23.	28.64	{9., 56.}	60.5217	0.244136	newly 3 weeks cumul	69.	63.81	{27., 112.}	41.8696	0.217	newly 5 weeks spec	34.	43.87	{13., 78.}	62.6176	0.273282	newly 5 weeks cumul	132.	144.36	{58., 260.}	47.5	0.216507	newly 62 weeks cumul	490.	689.32	{456., 841.}	47.2245	0.182671
8	newly 3 weeks spec	11.	29.06	{1., 60.}	200.727	0.532684	newly 3 weeks cumul	28.	56.08	{8., 117.}	131.286	0.361773	newly 5 weeks spec	39.	53.4	{2., 124.}	96.4615	0.547802	newly 5 weeks cumul	95.	150.56	{17., 351.}	106.442	0.405394	newly 66 weeks cumul	644.	664.96	{48., 913.}	35.7174	0.316008
9	newly 3 weeks spec	13.	39.77	{18., 63.}	206.231	0.440573	newly 3 weeks cumul	27.	87.6	{45., 135.}	224.444	0.477966	newly 5 weeks spec	24.	61.78	{29., 91.}	159.333	0.385724	newly 5 weeks cumul	73.	201.74	{103., 296.}	176.356	0.4104	newly 62 weeks cumul	503.	776.46	{668., 884.}	54.3658	0.18593
10	newly 3 weeks spec	19.	55.37	{34., 80.}	191.842	0.443721	newly 3 weeks cumul	44.	119.51	{78., 158.}	172.023	0.41691	newly 5 weeks spec	47.	81.02	{60., 102.}	74.1702	0.234992	newly 5 weeks cumul	144.	269.26	{192., 351.}	88.5972	0.267239	newly 62 weeks cumul	637.	836.27	{775., 890.}	31.2826	0.117496
11	newly 3 weeks spec	13.	31.16	{13., 50.}	145.846	0.367316	newly 3 weeks cumul	42.	75.53	{42., 109.}	85.9286	0.259284	newly 5 weeks spec	27.	44.01	{20., 67.}	77.0741	0.269357	newly 5 weeks cumul	89.	156.93	{81., 235.}	84.5056	0.261933	newly 59 weeks cumul	567.	628.35	{382., 774.}	28.2205	0.148399
12	newly 3 weeks spec	17.	18.03	{0., 45.}	114.176	0.828498	newly 3 weeks cumul	30.	30.57	{2., 79.}	100.967	0.561142	newly 5 weeks spec	56.	36.32	{0., 120.}	82.3571	0.972984	newly 5 weeks cumul	124.	94.63	{2., 267.}	90.5242	0.734674	newly 67 weeks cumul	637.	640.23	{2., 921.}	42.5133	0.517088
13	newly 3 weeks spec	11.	22.84	{7., 39.}	122.182	0.334652	newly 3 weeks cumul	31.	50.02	{20., 80.}	76.3871	0.241451	newly 5 weeks spec	30.	37.03	{13., 60.}	53.3	0.234274	newly 5 weeks cumul	95.	117.01	{50., 189.}	49.3789	0.19837	newly 62 weeks cumul	650.	715.1	{513., 837.}	20.5969	0.10307
14	newly 3 weeks spec	13.	8.28	{0., 20.}	98.3077	0.962603	newly 3 weeks cumul	26.	16.29	{1., 32.}	88.9615	0.726949	newly 5 weeks spec	24.	16.58	{0., 57.}	100.667	1.08925	newly 5 weeks cumul	70.	44.6	{2., 111.}	92.2857	0.817545	newly 67 weeks cumul	660.	453.32	{2., 901.}	54.2	0.842962
15	newly 3 weeks spec	34.	36.46	{6., 62.}	52.6471	0.343944	newly 3 weeks cumul	78.	96.98	{33., 152.}	53.2564	0.225132	newly 5 weeks spec	40.	38.22	{2., 69.}	49.8	0.422331	newly 5 weeks cumul	154.	174.33	{42., 278.}	48.3571	0.241468	newly 61 weeks cumul	487.	435.36	{116., 723.}	38.0903	0.258334
16	newly 3 weeks spec	11.	31.49	{1., 64.}	225.727	0.576261	newly 3 weeks cumul	21.	56.73	{5., 110.}	201.762	0.450824	newly 5 weeks spec	31.	59.95	{0., 146.}	147.129	0.667066	newly 5 weeks cumul	74.	164.44	{9., 340.}	165.189	0.495359	newly 67 weeks cumul	620.	651.97	{13., 912.}	41.6726	0.379942
17	newly 3 weeks spec	34.	41.68	{1., 97.}	96.2353	0.595654	newly 3 weeks cumul	57.	77.72	{8., 162.}	99.7193	0.434455	newly 5 weeks spec	41.	72.21	{1., 163.}	130.902	0.624334	newly 5 weeks cumul	126.	207.81	{15., 469.}	119.262	0.484331	newly 67 weeks cumul	668.	713.18	{50., 921.}	34.9521	0.316877
18	newly 3 weeks spec	10.	6.62	{0., 17.}	88.	0.841875	newly 3 weeks cumul	22.	12.54	{1., 31.}	75.8182	0.62339	newly 5 weeks spec	22.	17.8	{0., 59.}	97.6364	0.914649	newly 5 weeks cumul	59.	41.96	{2., 105.}	87.1186	0.680413	newly 67 weeks cumul	630.	532.47	{3., 908.}	49.6079	0.685472
19	newly 3 weeks spec	31.	27.08	{6., 42.}	50.9677	0.398606	newly 3 weeks cumul	69.	72.87	{24., 120.}	49.029	0.227838	newly 5 weeks spec	48.	26.67	{0., 51.}	52.3542	0.596094	newly 5 weeks cumul	148.	127.36	{39., 198.}	43.7838	0.261248	newly 60 weeks cumul	633.	428.81	{70., 652.}	33.7899	0.307017
20	newly 3 weeks spec	33.	36.47	{22., 53.}	29.7273	0.123202	newly 3 weeks cumul	87.	84.91	{54., 112.}	21.4368	0.0994785	newly 5 weeks spec	54.	52.94	{31., 74.}	22.963	0.10873	newly 5 weeks cumul	182.	182.73	{120., 241.}	21.511	0.0983291	newly 60 weeks cumul	622.	748.51	{652., 820.}	21.0788	0.0818669
21	newly 3 weeks spec	27.	83.16	{11., 166.}	229.481	0.573034	newly 3 weeks cumul	47.	159.07	{26., 302.}	255.085	0.532899	newly 5 weeks spec	26.	102.68	{28., 169.}	309.077	0.677081	newly 5 weeks cumul	97.	362.93	{73., 608.}	287.598	0.601531	newly 67 weeks cumul	609.	689.57	{360., 920.}	38.2644	0.251691
22	newly 3 weeks spec	20.	15.8	{2., 28.}	61.7	0.426075	newly 3 weeks cumul	51.	37.49	{9., 61.}	56.1765	0.352013	newly 5 weeks spec	65.	20.28	{2., 40.}	69.5385	0.756865	newly 5 weeks cumul	155.	75.83	{17., 130.}	61.8	0.502294	newly 61 weeks cumul	627.	490.31	{31., 740.}	32.6683	0.322198
23	newly 3 weeks spec	22.	45.87	{0., 83.}	138.136	0.556659	newly 3 weeks cumul	61.	123.45	{32., 186.}	120.082	0.354878	newly 5 weeks spec	31.	42.6	{0., 84.}	103.161	0.773832	newly 5 weeks cumul	113.	208.86	{38., 352.}	108.867	0.364075	newly 63 weeks cumul	543.	429.77	{38., 799.}	49.8508	0.40199
24	newly 3 weeks spec	19.	44.79	{19., 69.}	145.105	0.398007	newly 3 weeks cumul	60.	129.15	{63., 195.}	121.083	0.331488	newly 5 weeks spec	37.	39.79	{12., 64.}	43.1081	0.266788	newly 5 weeks cumul	115.	212.93	{97., 314.}	94.3739	0.28755	newly 60 weeks cumul	485.	410.22	{173., 625.}	30.5237	0.189702
25	newly 3 weeks spec	31.	16.25	{0., 35.}	70.3548	0.733463	newly 3 weeks cumul	67.	41.	{8., 87.}	62.9552	0.469428	newly 5 weeks spec	70.	19.16	{0., 45.}	74.9143	1.0276	newly 5 weeks cumul	176.	78.2	{11., 177.}	65.75	0.591729	newly 58 weeks cumul	653.	323.61	{15., 636.}	51.536	0.581083
26	newly 3 weeks spec	28.	15.58	{1., 27.}	68.2143	0.6376	newly 3 weeks cumul	57.	30.59	{4., 55.}	67.3158	0.546154	newly 5 weeks spec	43.	28.11	{1., 70.}	62.7674	0.616094	newly 5 weeks cumul	130.	79.66	{9., 169.}	62.6615	0.511727	newly 65 weeks cumul	587.	645.6	{23., 872.}	40.5826	0.340442
27	newly 3 weeks spec	19.	91.13	{55., 127.}	379.632	0.661267	newly 3 weeks cumul	36.	194.1	{114., 264.}	439.167	0.713112	newly 5 weeks spec	37.	109.9	{91., 129.}	197.027	0.467842	newly 5 weeks cumul	94.	414.47	{294., 524.}	340.926	0.634013	newly 64 weeks cumul	546.	835.39	{748., 896.}	53.0018	0.183661
28	newly 3 weeks spec	3.	14.75	{0., 35.}	447.	0.766067	newly 3 weeks cumul	9.	29.25	{2., 68.}	265.	0.51687	newly 5 weeks spec	27.	27.82	{0., 98.}	111.63	1.1165	newly 5 weeks cumul	65.	77.55	{2., 239.}	113.031	0.65788	newly 67 weeks cumul	649.	489.48	{4., 864.}	48.9861	0.749517
29	newly 3 weeks spec	26.	31.35	{14., 48.}	42.6538	0.16975	newly 3 weeks cumul	77.	72.94	{41., 100.}	26.2338	0.126528	newly 5 weeks spec	43.	46.75	{23., 71.}	35.6977	0.163127	newly 5 weeks cumul	154.	160.29	{92., 222.}	28.5649	0.128464	newly 60 weeks cumul	551.	700.37	{518., 815.}	32.7641	0.130103
30	newly 3 weeks spec	38.	26.05	{0., 61.}	57.2368	0.618013	newly 3 weeks cumul	100.	79.83	{18., 150.}	51.95	0.337683	newly 5 weeks spec	52.	21.38	{0., 45.}	63.1154	1.035	newly 5 weeks cumul	190.	124.51	{24., 269.}	49.7421	0.387074	newly 61 weeks cumul	601.	256.7	{24., 484.}	57.98	0.587705
31	newly 3 weeks spec	14.	34.75	{12., 60.}	157.071	0.387074	newly 3 weeks cumul	56.	79.65	{36., 140.}	60.9821	0.215351	newly 5 weeks spec	13.	47.27	{14., 81.}	271.923	0.548233	newly 5 weeks cumul	98.	168.88	{67., 285.}	86.2041	0.269324	newly 62 weeks cumul	505.	632.28	{395., 821.}	39.5802	0.18933
32	newly 3 weeks spec	13.	27.7	{6., 54.}	134.154	0.370217	newly 3 weeks cumul	24.	55.84	{18., 111.}	143.417	0.343135	newly 5 weeks spec	8.	51.43	{9., 105.}	550.375	0.728218	newly 5 weeks cumul	57.	146.31	{40., 276.}	168.158	0.379563	newly 64 weeks cumul	509.	787.56	{632., 918.}	57.1434	0.20272
33	newly 3 weeks spec	19.	39.15	{3., 108.}	155.421	0.476631	newly 3 weeks cumul	30.	73.46	{9., 204.}	177.467	0.408049	newly 5 weeks spec	36.	62.82	{5., 137.}	118.833	0.485442	newly 5 weeks cumul	97.	189.47	{19., 523.}	134.361	0.42245	newly 67 weeks cumul	689.	679.68	{173., 924.}	33.344	0.259043
34	newly 3 weeks spec	23.	41.3	{8., 70.}	103.478	0.395	newly 3 weeks cumul	76.	121.84	{47., 188.}	77.6053	0.270181	newly 5 weeks spec	29.	30.1	{1., 55.}	54.6207	0.444975	newly 5 weeks cumul	132.	187.53	{60., 285.}	62.8258	0.254	newly 59 weeks cumul	459.	322.95	{75., 578.}	43.4314	0.328784
35	newly 3 weeks spec	15.	87.86	{17., 157.}	496.533	0.754539	newly 3 weeks cumul	32.	154.26	{32., 279.}	391.938	0.634356	newly 5 weeks spec	45.	128.43	{43., 181.}	197.622	0.549968	newly 5 weeks cumul	99.	405.54	{146., 634.}	320.242	0.612801	newly 67 weeks cumul	642.	794.2	{540., 932.}	35.433	0.189995
36	newly 3 weeks spec	67.	47.3	{1., 120.}	82.1194	0.792904	newly 3 weeks cumul	106.	86.27	{7., 238.}	88.5189	0.624442	newly 5 weeks spec	61.	73.4	{0., 161.}	93.0164	0.701303	newly 5 weeks cumul	222.	222.85	{13., 562.}	83.545	0.575545	newly 68 weeks cumul	649.	706.2	{18., 925.}	38.0123	0.347932
37	newly 3 weeks spec	27.	29.13	{11., 51.}	45.2222	0.217238	newly 3 weeks cumul	77.	77.28	{38., 124.}	36.6494	0.182811	newly 5 weeks spec	49.	33.45	{14., 57.}	41.0408	0.307666	newly 5 weeks cumul	165.	142.98	{70., 248.}	35.6727	0.205166	newly 58 weeks cumul	598.	471.77	{293., 696.}	28.2525	0.180554
38	newly 3 weeks spec	10.	68.24	{15., 140.}	585.8	0.733913	newly 3 weeks cumul	38.	126.64	{32., 238.}	241.263	0.467183	newly 5 weeks spec	51.	112.89	{47., 157.}	127.588	0.341394	newly 5 weeks cumul	123.	337.18	{116., 544.}	180.195	0.408912	newly 66 weeks cumul	597.	832.81	{654., 915.}	42.1189	0.152752
39	newly 3 weeks spec	24.	25.04	{8., 46.}	47.6667	0.227245	newly 3 weeks cumul	41.	58.96	{24., 100.}	64.1951	0.217502	newly 5 weeks spec	32.	35.32	{11., 64.}	52.4375	0.260234	newly 5 weeks cumul	105.	126.08	{48., 212.}	53.4286	0.219528	newly 62 weeks cumul	520.	645.99	{422., 799.}	33.7327	0.145073
40	newly 3 weeks spec	29.	12.69	{0., 32.}	84.0345	1.02997	newly 3 weeks cumul	61.	22.25	{1., 50.}	80.2131	0.839212	newly 5 weeks spec	48.	31.51	{0., 96.}	87.1042	1.05033	newly 5 weeks cumul	145.	73.56	{2., 201.}	80.4414	0.851892	newly 68 weeks cumul	580.	574.64	{2., 921.}	55.5448	0.695383
41	newly 3 weeks spec	22.	38.95	{9., 65.}	97.1364	0.291602	newly 3 weeks cumul	38.	74.91	{25., 123.}	109.868	0.298671	newly 5 weeks spec	81.	76.48	{22., 120.}	34.9136	0.20073	newly 5 weeks cumul	156.	210.77	{77., 341.}	58.7885	0.225064	newly 65 weeks cumul	649.	827.65	{703., 920.}	29.7488	0.113302
42	newly 3 weeks spec	18.	56.69	{18., 87.}	225.389	0.522638	newly 3 weeks cumul	47.	129.51	{58., 212.}	180.787	0.425975	newly 5 weeks spec	26.	75.71	{18., 117.}	209.423	0.600726	newly 5 weeks cumul	102.	273.69	{107., 400.}	175.637	0.431311	newly 65 weeks cumul	610.	670.36	{237., 897.}	38.318	0.218956
43	newly 3 weeks spec	35.	18.76	{9., 30.}	48.5714	0.351085	newly 3 weeks cumul	83.	44.59	{23., 65.}	47.8675	0.318514	newly 5 weeks spec	42.	24.25	{10., 40.}	44.4524	0.32253	newly 5 weeks cumul	155.	90.44	{48., 135.}	43.1871	0.284816	newly 58 weeks cumul	514.	601.52	{459., 715.}	23.5875	0.0980436
44	newly 3 weeks spec	15.	37.24	{5., 73.}	175.067	0.445998	newly 3 weeks cumul	34.	69.98	{13., 143.}	132.706	0.363007	newly 5 weeks spec	30.	76.05	{12., 132.}	175.567	0.466782	newly 5 weeks cumul	77.	203.16	{45., 374.}	182.961	0.431111	newly 66 weeks cumul	567.	793.13	{610., 923.}	48.1041	0.203278
45	newly 3 weeks spec	32.	54.47	{23., 83.}	83.5938	0.271075	newly 3 weeks cumul	68.	125.75	{60., 188.}	90.8382	0.255024	newly 5 weeks spec	56.	64.44	{26., 94.}	39.75	0.214071	newly 5 weeks cumul	159.	252.67	{117., 356.}	69.0881	0.224013	newly 63 weeks cumul	632.	655.83	{403., 835.}	23.5585	0.116405
46	newly 3 weeks spec	21.	18.75	{1., 40.}	59.2857	0.481002	newly 3 weeks cumul	37.	46.59	{11., 85.}	63.7568	0.268631	newly 5 weeks spec	19.	29.08	{0., 60.}	102.526	0.570931	newly 5 weeks cumul	68.	99.08	{15., 192.}	83.8824	0.336878	newly 62 weeks cumul	566.	524.68	{15., 767.}	37.7032	0.350726
47	newly 3 weeks spec	13.	52.97	{1., 144.}	360.385	0.717477	newly 3 weeks cumul	43.	97.43	{4., 230.}	402.143	0.572997	newly 5 weeks spec	26.	74.73	{0., 168.}	242.423	0.764513	newly 5 weeks cumul	64.	238.91	{9., 614.}	319.453	0.637837	newly 68 weeks cumul	615.	683.37	{15., 928.}	42.6618	0.375471
48	newly 3 weeks spec	37.	66.99	{34., 97.}	87.1622	0.260623	newly 3 weeks cumul	81.	151.6	{83., 220.}	91.2099	0.266117	newly 5 weeks spec	34.	85.	{57., 108.}	151.882	0.391864	newly 5 weeks cumul	153.	315.57	{181., 431.}	108.333	0.305724	newly 62 weeks cumul	483.	780.99	{657., 879.}	61.9979	0.206773
49	newly 3 weeks spec	13.	43.73	{6., 90.}	254.231	0.514841	newly 3 weeks cumul	42.	82.6	{19., 169.}	116.762	0.308198	newly 5 weeks spec	46.	78.76	{20., 135.}	93.913	0.326992	newly 5 weeks cumul	113.	224.77	{57., 406.}	117.938	0.33247	newly 66 weeks cumul	632.	807.96	{663., 918.}	34.6614	0.14344
50	newly 3 weeks spec	9.	22.09	{2., 37.}	174.111	0.460498	newly 3 weeks cumul	25.	45.61	{12., 72.}	105.4	0.292071	newly 5 weeks spec	19.	37.65	{5., 83.}	129.	0.419168	newly 5 weeks cumul	58.	113.91	{20., 213.}	122.224	0.336714	newly 64 weeks cumul	488.	688.28	{461., 852.}	50.8566	0.220135
